# PERCEIVED FEASIBILITY TO IMPLEMENT A TASK-ORIENTED EXERCISE PROGRAM BY FITNESS STAFF AT SENIOR LIVING COMMUNITIES

**DOI:** 10.1093/geroni/igad104.1724

**Published:** 2023-12-21

**Authors:** Chiung-ju Liu, Lauren Mansuy

**Affiliations:** University of Florida, Gainesville, Florida, United States; University of Central Florida, Orlando, Florida, United States

## Abstract

Older adults who relocate to senior living communities have a desire to maintain their independence. The fitness infrastructure within these communities is a great platform to deliver evidence-based exercise programs to support residents’ independence, especially for those who have experienced activity limitations. Task-oriented exercise incorporates daily activities into exercise strategies and has been recommended as an approach to reduce late-life disability. The current study used an interview research design to determine the feasibility of implementing 3-Step Workout for Life, a task-oriented program, in senior living communities. The 3-Step Workout for Life program consists of gym-based group resistance exercise and home-based one-on-one activity exercise. Fourteen fitness instructors from different communities, with independent living units, in the state of Florida completed the study. Interviews were transcribed, coded, and underwent thematic analysis. Instructors perceived the screening procedure to select and enroll residents in this program could help match residents to the right fitness program. The gym-based group resistance exercise is compatible with existing instructor-led fitness programs. Although instructors perceived the home-based one-on-one activity exercise positively, such as the potential benefits for frail residents who do not attend the gym, they acknowledged several barriers to implementing this exercise. Major barriers include their job responsibility restricting them from working in the resident’s home, their belief that activity exercise should be better delivered by a rehabilitation professional, and limited staff to support the one-on-one format. The next step is to collaborate with community stakeholders to address these barriers and build capacity to implement task-oriented exercise programs.

